# Complete Genome Sequence of *Bacillus altitudinis* P-10, a Potential Bioprotectant against *Xanthomonas oryzae* pv. oryzae, Isolated from Rice Rhizosphere in Java, Indonesia

**DOI:** 10.1128/genomeA.01388-17

**Published:** 2017-11-30

**Authors:** Anto Budiharjo, Haeyoung Jeong, Dyah Wulandari, Soohyun Lee, Choong-Min Ryu

**Affiliations:** a Biology Department, Faculty of Sciences and Mathematics, Diponegoro University, Semarang, Indonesia; b Infectious Disease Research Center, Korea Research Institute of Bioscience and Biotechnology (KRIBB), Daejeon, Republic of Korea; c School of Biotechnology, Institute of Agricultural Technology, Suranaree University of Technology, Ratchasima, Thailand

## Abstract

*Bacillus altitudinis* P-10 was isolated from the rhizosphere of rice grown in an organic rice field and provides strong antagonism against the bacterial blight caused by *Xanthomonas oryzae* pv. oryzae in rice. Herein, we provide the complete genome sequence and a possible explanation of the antibiotic function of the P-10 strain.

## GENOME ANNOUNCEMENT

The species *Bacillus altitudinis* and three other novel species (*B. aerius*, *B. aerophilus*, and *B. stratosphericus*) were isolated and characterized from air samples in 2006 (1). This species was later recognized as an enzyme producer with potential industrial uses (2–6) and as a plant growth-promoting rhizobacterium (7). In addition, endophytic *B. altitudinis* isolated from the roots of healthy wheat showed the ability to bioleach iron, an important feature for lightening iron toxicity in wheat (8). However, because its 16S rRNA gene sequence is highly similar to those of *B. pumilus* group species, such as *B. pumilus*, *B. safensis*, *B. stratosphericus*, *B. altitudinis*, and *B. aerophilus* (9), a multilocus sequence analysis or a genome sequence-based analysis is required for a study of the diversity and phylogeny of this group.

*B. altitudinis* strain P-10 was isolated from the rhizosphere of rice in an organic rice farm in the Banyubiru District, Semarang Regency, central Java Province, Indonesia (10). Instead of a chemical fertilizer, a microbe-based biofertilizer has been applied to this rice field for a long period of time. The farmers use a biofertilizer solely to cultivate crops on this farm, because the soil, generated from an ash component created through volcanic activity, does not contain enough nutrients for bacterial growth. *B. altitudinis* strain P-10 displays direct antagonism against *Xanthomonas oryzae* pv. oryzae, a casual pathogen of rice bacterial blight, which has caused significant yield loss in Asian counties (10).

Genome sequencing was carried out on a PacBio RSII platform from a single-molecule real-time (SMRT) cell using P6-C4 chemistry at the Theragen Etex Bio Institute (Suwon, Gyeonggi-do, Republic of Korea). *De novo* assembly using the RS_HGAP_Assembly.2 protocol under SMRT Analysis 2.3.0 produced one contig from 1.27 Gb of raw sequencing reads, followed by circularization using Circlator (11). Residual errors were corrected by running two successive rounds of the RS_Resequencing.1 protocol, followed by additional correction through the mapping of corrected long reads in the CLC Genomics Workbench (Qiagen Bioinformatics, Aarhus, Denmark). The complete genome sequence was annotated using NCBI’s Prokaryotic Genome Annotation Pipeline and the Rapid Annotation of microbial genomes using Subsystems Technology (RAST) server (12).

The P-10 genome has a 3,765,752-bp chromosome (41.5% G+C content) containing 3,828 protein-coding genes, 81 tRNA genes, and 8 rRNA operons, with no plasmids. Although initially classified as *B. pumilus* based on the 16S rRNA gene sequence analysis, the Genome-to-Genome Distance Calculator (http://ggdc.dsmz.de/ggdc.php#) indicates that strain P-10 belongs to the *B. altitudinis* species, where the DNA-DNA hybridization (DDH) estimate with *B. altitudinis* 41KF2b^T^ (GenBank accession number ASJC00000000) is 86.4%.

antiSMASH 3.0 (13) predicted six biosynthetic gene clusters for lichenysin- or bacilysin-like nonribosomal peptides, terpenes, and bacteriocin. Because no notable pathogenicity-related genes have been found from Island Viewer analysis (14), *B. altitudinis* strain P-10 can be considered a promising candidate for bioprotectant development. In conclusion, the complete genome sequence of strain P-10 presents a beneficial trait and a possible explanation of its antibiotic function.

### Accession number(s).

The complete genome sequence of *B. altitudinis* P-10 has been deposited in DDBJ/ENA/GenBank under the accession number CP024204.
